# Blast Test and Failure Mechanisms of Soft-Core Sandwich Panels for Storage Halls Applications

**DOI:** 10.3390/ma14010070

**Published:** 2020-12-25

**Authors:** Robert Studziński, Tomasz Gajewski, Michał Malendowski, Wojciech Sumelka, Hasan Al-Rifaie, Piotr Peksa, Piotr W. Sielicki

**Affiliations:** 1 Institute of Building Engineering, Faculty of Civil and Transport Engineering, Poznan University of Technology, 61-614 Poznań, Poland; 2 Institute of Structural Analysis, Faculty of Civil and Transport Engineering, Poznan University of Technology, 61-614 Poznań, Poland; tomasz.gajewski@put.poznan.pl (T.G.); michal.malendowski@put.poznan.pl (M.M.); wojciech.sumelka@put.poznan.pl (W.S.); hasan.al-rifaie@put.poznan.pl (H.A.-R.); piotr.peksa@put.poznan.pl (P.P.); piotr.sielicki@put.poznan.pl (P.W.S.)

**Keywords:** sandwich panel, explosive, blast load, failure mechanisms, continuous core, non-continuous core, fasteners

## Abstract

In this paper, an experimental investigation is presented for sandwich panels with various core layer materials (polyisocyanurate foam, mineral wool, and expanded polystyrene) when subjected to a justified blast load. The field tests simulated the case for when 5 kg of trinitrotoluene (TNT) is localized outside a building’s facade with a 5150 mm stand-off distance. The size and distance of the blast load from the obstacle can be understood as the case of both accidental action and a real terroristic threat. The sandwich panels have a nominal thickness, with the core layer equal 100 mm and total exterior dimensions of 1180 mm × 3430 mm. Each sandwich panel was connected with two steel columns made of I140 PE section using three self-drilling fasteners per panel width, which is a standard number of fasteners suggested by the producers. The steel columns were attached to massive reinforced concrete blocks via wedge anchors. The conducted tests revealed that the weakest links of a single sandwich panel, subjected to a blast load, were both the fasteners and the strength of the core. Due to the shear failure of the fasteners, the integrity between the sandwich panel and the main structure is not provided. A comparison between the failure mechanisms for continuous (polyisocyanurate foam and expanded polystyrene) and non-continuous (mineral wool) core layer materials were conducted.

## 1. Introduction

The composite structures considered in this paper consist of three components: two external facings and an internal core. The facings are made of high-strength steel while the core is made of a low-strength, soft material with excellent thermal insulation properties, namely polyisocyanurate (PIR) foam, mineral wool, or expanded polystyrene. This type of composite structure is used as the roof or wall cladding elements of storage halls, magazines, or cold storage halls. In these applications, the thickness of the facings varies between 0.4 mm and 1.0 mm, while the core ranges between 40 mm and 200 mm. During the assumed service life of sandwich panels, they are subjected to permanent, variable, and accidental actions. According to European standard EN 1990 [1], permanent actions (self-weight, or the weight of the infrastructural elements) are likely to act throughout the assumed service life. The variable actions (wind load, snow load, and temperature gradient) are characterized by variation in their magnitudes with time. The accidental actions, which are unlikely to occur (fire, impact, exceptional snow fail, seismic action, and explosion) are of short durations but significant magnitudes. The wide use of sandwich panels in industrial construction increases the importance of accidental actions, due to the presence of industrial equipment, such as oxygen tanks, gas cylinders, and high-pressure tanks. The above-mentioned infrastructural elements raise the risk of an explosion, which is influenced by their size and location in relation to surrounding sandwich panels (as described later in Section 2).

The latest recommendations for the application of sandwich panels were briefly presented in [2], based on the European standard EN 14,509 [3]. The EN 14,509 standard provides detailed information about the influence of openings on the capacity of sandwich panels and the possibility of lateral stabilization using beams, purlins, and side rails. The phenomenon of stabilization of compressed or bent thin-walled elements by the sandwich panels was thoroughly investigated in [4,5,6]. However, other researchers highlighted that the problem is still an open question [7,8,9]. In [10], the use of the composite sandwich panels was discussed alongside following the modern requirements of building energy-saving and even the adaptation of housing industrialization. To the authors’ knowledge, the response of the sandwich panels (used in structural engineering applications) to blast threats are not well covered in the literature.

Over the last few decades, extensive research was conducted to provide knowledge about the impact of explosions on the structural integrity and safety of buildings. Starting from the early 1970s, the measurements of relevant explosion parameters needed for impact assessment of gas explosions in buildings were considered in [11]. These explosion parameters were obtained from the built explosion chamber of a volume of 28.4 m^3^. At the same time, in [12], the factors affecting both the development and the severity of the explosions in large compartments were presented. Additionally, the aspect of venting on the relieving of explosion pressure was undertaken. In the late 1990s, in [13], the pressure–time behavior of vented buildings was simulated with the use of the lumped parameter model. Using this model, real explosions in residential structures and industrial plants were modelled. Recently, in [14], attention was paid to beam-to-beam and beam-to-column connections, described as the weakest links of multistory buildings subjected to blasts. The multistory steel frame structures subjected to explosions were also discussed in [15], where both the robustness curves and problem of precise evaluation of the blast pressure by means of computational fluid mechanics (CFD) methods were presented. Another example of the impact of an explosion on a steel structure can be found in [16], where the case of a close-in blast loading on a metallic obstacle (column made of S355 of an HKS 300-4 section) was modelled in terms of the generalized thermo-elasto-viscoplastic (GTEV) approach. Moreover, the blast resistance of large steel gates equipped with uniaxial graded auxetic dampers (UGAD) was presented in [17]. Detailed information about the shock-absorbing uniaxial graded auxetic dampers can be found in [18]. Masonry structures are also vulnerable to blast loads. In [18], the experimental and numerical investigation of masonry wall behavior under blast loading was discussed. Additionally, in [19], the safety conditions for a one-way masonry wall under a blast, and the numerical solution for coupled charge–air–structure integration inducing failure, were proposed. The research papers presented above deal with the behavior of the building (as a whole) or with a specific structural element when subjected to accidental actions caused by the explosion.

On the other hand, metallic sandwich panels (composed of an aluminum core sandwiched between two plates) are well covered in the literature. They are used as lightweight energy absorbers to mitigate shock, blast, and impact cases. Their applications range from lightweight transportation solutions (aircraft, high-speed trains, and ships) to other protective structures. The composite sandwich panels used in the construction of navy ships are subjected to several threats, such as low-velocity impacts [20], dynamic transverse impacts [21], air blast loading [22,23], and underwater blasts [24,25]. In [20], first the term low-velocity impact is provided, and then the following low-velocity failure mechanisms are described: matrix damage, delamination, fiber failure, and penetration. Additionally, the influence of the fibers and the interphase region are discussed. In [21], the dynamic response of glass fiber-vinylester composite beams was presented. The aim of the paper [21] was to investigate the dynamic deformation and failure mechanisms with the understanding of the influence of the mixed core compositions from the following materials: H100 PVC, Balsa, and H250 PVC. In [22], the failure mode maps of the sandwich panels subjected to air blast loading were provided. Note that in [22], the dynamic effect was modeled as a single-degree-of-freedom mass–spring system. On the contrary, the researchers’ efforts in the case of the composite sandwich structures used for protection purposes (i.e., the structures subjected to air blasts or impact loading) focused on finding the optimal core solutions. The following sandwich panels cores’ conceptions were investigated over the years: cores made of aluminum honeycomb [26,27], crosslinked polyvinyl chloride (PVC) foam [28], reinforced PVC foam [29], polyurethane (PUR) foam [30], corrugated cores filled with aluminum foam [31], and thermoplastic polyurethane (TPU) foam [32]. Note that in [30], the failure mechanisms were obtained by means of simple drop weight tests. The various impact energies, ordering of the layers, and core densities were investigated. In [31], the metallic corrugated core sandwich panels subjected to air blast loading were presented. In the field tests, explosives of a mass of 55 g were used. Two distances between the charge and the external layer of the sandwich panels were investigated, specifically 50 mm and 100 mm, in order to find the mechanical and kinematical responses of three core concepts, namely the aluminum foam-filled corrugated core, empty corrugated core, and aluminum core.

This study investigates the qualitative response and the failure mechanisms of full-scale sandwich panels, with various core layers subjected to a specific blast load of 5 kg of TNT at a 5150 mm stand-off distance. The size and distance of the blast load from the obstacle can be understood as the case of both the accidental action and the real terroristic threat. The stand-off distance is counted from the explosive centroid to the external facing of the panel. The after blast deformations of the sandwich panels are described in detail. To the authors’ knowledge, the presented problem is original and has not been investigated before.

## 2. Materials and Methods

In this paper, full-scale experiments of sandwich panels subjected to blast loading are presented, which can be classified as a free air (spherical) unconfined explosion (i.e., an explosion occurred in free air where a shock wave propagates without intermediate amplification). Note that the assumed distance was not a close-in detonation. The nominal thickness of all tested panels was 100 mm. This thickness of the sandwich panel’s inner core is the key capacity factor which controls the withstanding wind pressure and suction. The sandwich panels were of 1180 mm in width and 3430 mm in length. Two types of core layer were investigated, namely the continuous core (polyisocyanurate foam), also referred to as PIR foam, and expanded polystyrene and the non-continuous core (mineral wool). According to the declarations of the producer of the sandwich panels (Pruszyński Sp. z o.o., Sokołów, Poland) used in the experiment, the characteristic values of the shear modulus *G_C_* for the PIR foam, mineral wool, and expanded polystyrene were 3.2 MPa [33], 1.9 MPa [34], and 1.5 MPa [35], respectively. The sandwich panel external facings were made of zinc-coated steel of a grade S280GD and of a nominal thickness of 0.5 mm. The tensile Young’s modulus *E* of the facings was 186,000 MPa [36]. Since the Young’s modulus of the facings was 3 × 10^4^ times larger than the Young’s modulus of the core, the normal stresses were transferred by the stiff facings, while the shear stresses were transferred by the softcore. For more information about the computational models and theories of the sandwich panels, refer to the state-of-the-art review article [37].

**Figure 1:**
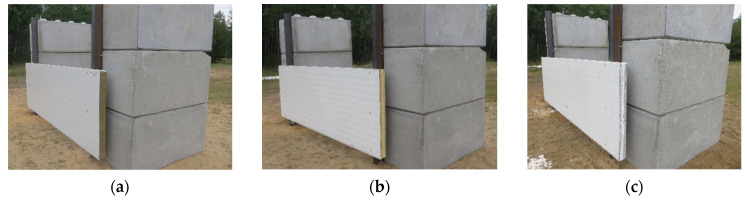
View of the field test setup, showing the installed sandwich panel with the core made of the different core materials: (**a**) mineral wool; (**b**) polyisocyanurate foam; and (**c**) expanded polystyrene.

The field test setup consisted of six massive reinforced concrete blocks and two I140 PE steel columns (Konsorcjum Stali S.A., Oddział Poznań, Poznan, Poland). Six of these reinforced concrete (RC) blocks (MM-Trans, Poznan, Poland) of a cuboid shape (width 800 mm, length 1600 mm, and height 800 mm) were used as supports (3 on each side, Figure 1). 

**Figure 2:**
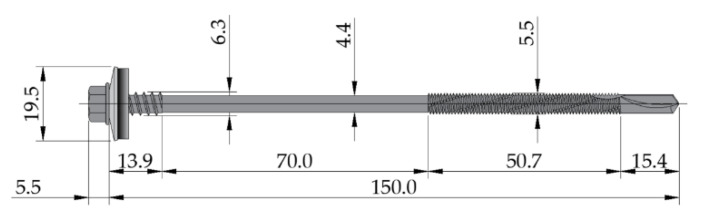
The geometry of the self-drilling fastener (dimensions are in mm).

The I140 PE columns were 3000 mm in length and made of S235 steel. They were attached to the massive blocks via the wedge anchors and dug into the ground at a depth of 600 mm. Then, each sandwich panel was attached to the outstanding flange of the I140 PE columns via the self-drilling fasteners (three fasteners per width) made of carbon steel (grade S280 GD) with an ethylene propylene diene monomer rubber (EPDM) sealing washer with a metal top (see Figure 2). The three fasteners per panel width were recommended by the producer’s number of fasteners.

**Figure 3:**
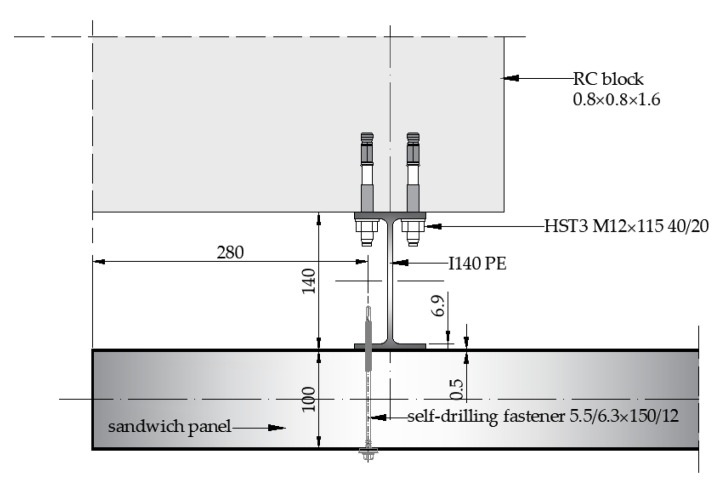
Detail of the connection of the sandwich panel with the I140 PE column (dimensions are in mm).

According to the European Technical Assessment [38], the characteristic shear and tension capacity of the used fasteners for the thicknesses of the connected elements (0.5 mm external facing, 6.9 mm I140 PE flange; see Figure 3) were equal to *V_R,k_* = 1.73 kN and *N_R,k_* = 3.75 kN, respectively.

**Figure 4:**
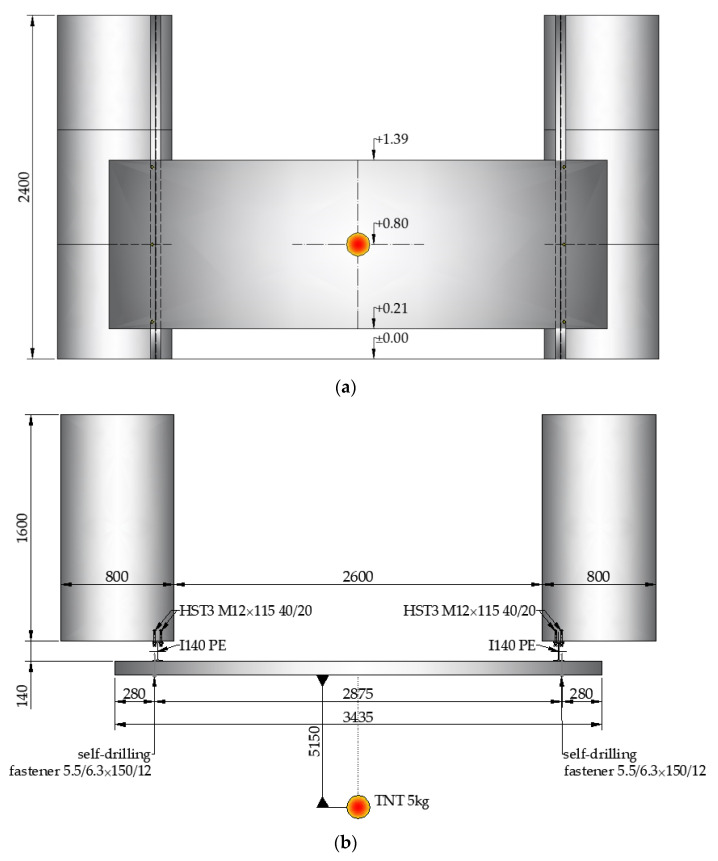
Scheme of the field test (dimensions are in mm): (**a**) front view and (**b**) top view.

In Figure 4, the scheme of the test setup with the location of the explosive (5 kg of TNT) is presented. The explosive centroid was positioned at a height of 800 mm from the ground with a horizontal stand-off distance of 5150 mm. The field test setup with the installed sandwich panel is presented in Figure 1.

Table 1 depicts the selected industrial infrastructural elements, which could be localized outside the building, and provides estimated information about their maximum pressures developed at completion of combustion (*P_max_*), blast wave energy (*E*), and calculated equivalent TNT mass (*W_TNT_*).

The calculations presented in Table 1 were based on principles developed in [39]. The following input parameters, including the explosive fuels information, were assumed:

- Ambient air temperature *T_a_* = 25 °C;
- Initial (default) atmospheric pressure *P_s_* = 101.35 kPa;
- The fraction of available combustion for unconfined mass release *α* = 1%;
- Adiabatic flame temperature of the propane *T_ad_*(propane) = 1281 °C;
- Adiabatic flame temperature of the acetylene *T_ad_*(acetylene) = 2637 °C;
- Adiabatic flame temperature of the hydrogen *T_ad_*(hydrogen) = 2252 °C;
- The heat of combustion of the propane Δ*H_c_*(propane) = 46,360 kJ/kg;
- The heat of combustion of the acetylene Δ*H_c_*(acetylene) = 48,220 kJ/kg;
- The heat of combustion of the hydrogen Δ*H_c_*(hydrogen) = 130,800 kJ/kg.

The maximum pressure developed at the completion of combustion, the blast wave energy, and the equivalent TNT mass were expressed by Equations (1)–(3), respectively:

(1) $$P_{max}\;=\;\frac{T_{ad}}{T_{a}}P_{a},$$

(2) $$E\;=\;\alpha \times \Delta H_{c}\times m_{F},$$

(3) $$W_{TNT}\;=\;\frac{E}{4500}.$$

Note that in Table 1, the equivalent TNT masses, which were close to the mass of explosives used in the experiment at the military training ground, are bolded.

**Figure 5:**
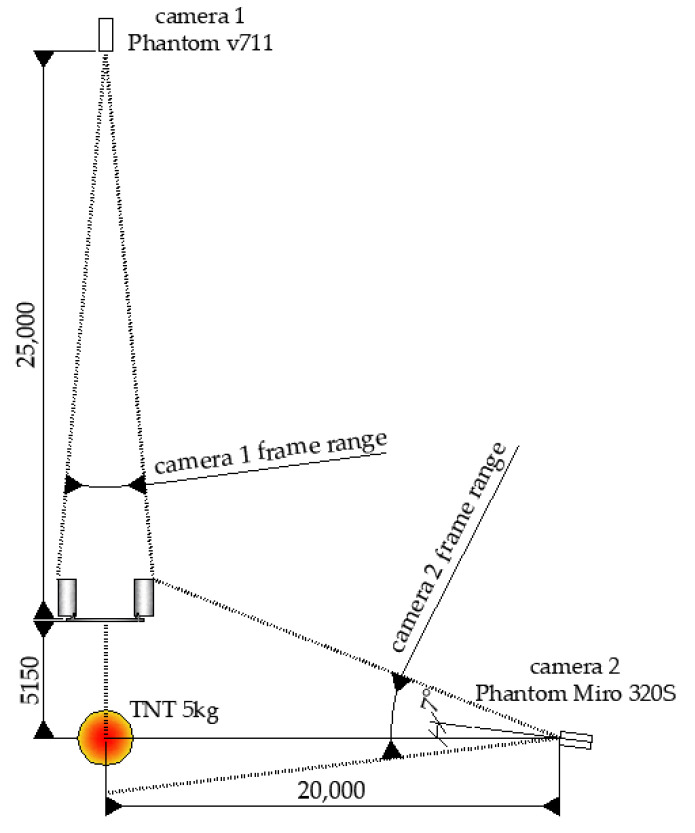
The positions of the cameras in relation to the tested structure (dimensions are in mm).

During the field tests, two high-speed cameras were used. The position of the cameras in relation to the tested structure is shown in Figure 5. Camera 1 (Phantom v711, Vision Research, Inc., Wayne, NJ, USA), located behind the sandwich panel, recorded the tests with a speed of 1000 fps (frames per second) and with an 800 × 432 resolution. Camera 2 (Phantom Miro 320S, Vision Research, Inc., Wayne, NJ, USA), located at the side of the sandwich panel, recorded the tests with a speed of 1000 fps (frames per second) and with a 1280 × 632 resolution.

## 3. Results

The presentation of the results from the field tests is divided into two stages. First, the analytical approach allowing the determination of the explosion pressure distribution on the external sandwich panel facing will be presented. Then, the sequence of the failure mechanisms obtained during the field tests is shown and discussed. Note that the blast load acting on the sandwich panels during the field tests can be classified as a free air (spherical) unconfined explosion (i.e., an explosion occurred in open air, where a shock wave propagates without intermediate amplification).

### 3.1. Analytical Approach

**Figure 6:**
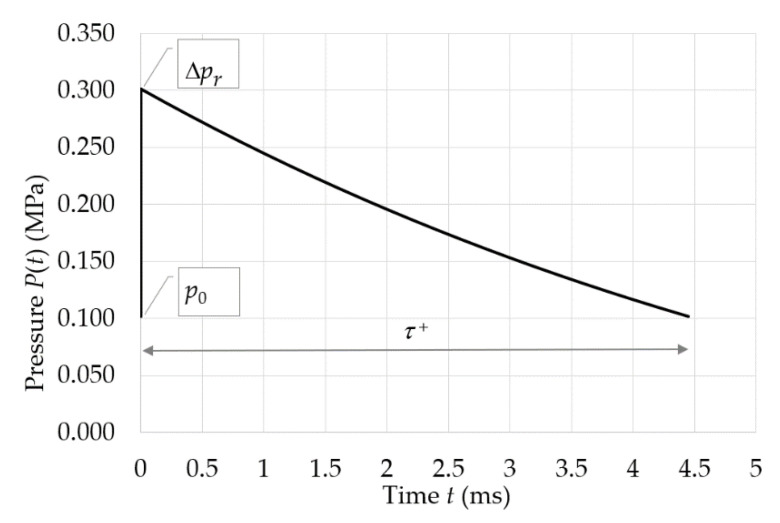
Variation of the overpressure with respect to time of the middle point of the sandwich panel’s external facing.

The load on the surfaces of a building caused by an explosion depends on the detonation products. The pressure during an explosion intensifies rapidly and leads to an increase in the density and temperature of the medium. These create pressure waves, which have the nature of shock waves. There are two ways to take into account the impact of an explosion on the facades. The first one requires the determination of the shock wave pulse, while the other one, which was implemented in this article, requires determination of the pressure–time function (see Figure 6). In Figure 6, the pressure–time diagram for the middle point at the external facing of the sandwich panel (see Equation (9)) is plotted, where Δ*P_r_* represents the overpressure at the wavefront, *p*_0_ represents the initial pressure in the medium (from the International Standard Atmosphere (ISA) model), and *τ*^+^ represents the duration of the overpressure. The results depicted in Figure 6 are in agreement with the results obtained from the software for the rapid prediction of blast wave properties [40] (i.e., Δ*p_r_* = 0.21 MPa (+4.8% difference) and *τ*^+^ = 4.60 ms (+3.3% difference)).

The algorithm used to determine the explosive load caused by the detonation of the external charge of 5 kg of TNT is presented below. The equivalent charge radius *r*_0_ = 6.05 m was obtained according to Sachs’ law [41,42] from Equation (4):

(4) $$r_{0}\;=\;{\left(\frac{E}{p_{0}}\right)}^{\frac{1}{3}}$$

where *p*_0_ represents the initial pressure in the medium, taken from the International Standard Atmosphere model (ISA) (*p*_0_ = 101,325 Pa ≅ 0,1 MPa); *E* = *mQ* represents the explosion energy; *m* represents the charge mass (*m* = 5 kg); and *Q* represents the heat of the explosion for the TNT (*Q* = 4500 kJ/kg) [19].

The proximity factor *Z*, expressed by Equation (5), indicates the two explosion zones, namely the close zone and the far zone, for *Z* ≤ 1.0 and *Z* > 1.0, respectively.

(5) $$Z\;=\;\frac{r}{\sqrt[{3}]{m}}$$

where *r* represents the distance from the center of the spherical charge. In this study, *r* = 5.15 m was the middle point of the sandwich panel’s external facing.

In our case, *Z* = 3.01 m/kg^1/3^, which means this was the far zone case. Having the proximity factor *Z*, the nondimensional overpressure *p_s_* = 0.76 was obtained from Equation (6), proposed by Sadovskiy [43]:

(6) $$p_{s}\;=\;\frac{0.754}{Z}+\frac{2.457}{Z^{2}}+\frac{6.5}{Z^{3}}$$

Having the nondimensional overpressure *p*_s_, the peak reflected overpressure Δ*p_r_* could be obtained from the Rankine–Hugonoit relationship for an ideal gas [44], as shown in Equation (7):

(7) $$\Delta p_{r}\;=\;2\Delta p_{s}+\frac{6\Delta p_{s}^{2}}{\Delta p_{s}+7p_{0}}$$

where Δ*p_s_* = *p_s_*⋅*p*_0_ = 0.76⋅0.1 = 0.08 MPa is the free air blast overpressure.

In our case, Δ*p_r_* = 0.20 MPa for the middle point of the sandwich panel’s external facing. The duration of the blast wave *τ*^+^, which is defined as the time difference between the passing of the shock wave’s front and the passing of the end of the positive pressure phase, can be obtained. When the proximity factor *Z* is in the range (1, 15), the duration of the blast wave (*τ*^+^ = 4.45 ms) can be defined in accordance to Sadovskiy’s proposal [43], shown in Equation (8):

(8) $$\tau^{+}\;=\;1.5\sqrt{Z}\times \sqrt[{3}]{m}$$

Finally, the change in time of the overpressure Δ*p*(*t*) is expressed by the Liu and Chiu formula [45], shown in Equation (9):

(9) $$\Delta p\left(t\right)\;=\;\Delta p_{r}\left(1-\frac{t}{\tau^{+}}\right)e^{\left(-\frac{at}{\tau^{+}}\right)}\;and\;a\;=\;1.39\;\;\Delta p_{s}^{0.54}$$

**Figure 7:**
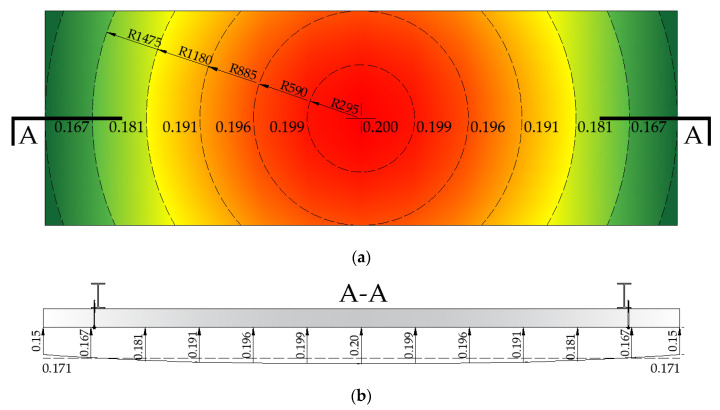
Variation of the overpressure Δ*p_r_* in MPa on the front surface of a sandwich panel. (**a**) Front view. (**b**) Cross-section A-A view.

The change in time of the overpressure Δ*p*(*t*) for the middle point of the sandwich panel is presented in Figure 6. The distribution of both the maximal overpressure Δ*p_r_* in MPa and the mean overpressure (represented by the dashed line) Δ*p_r,mean_* = 0.171 MPa on the front surface of the sandwich panel are presented in Figure 7. Note that the presented maximal overpressure cannot be directly used as the load in the design. Instead, the time-varying equivalent pressure, which depends on the equivalent factor, as well as the drag coefficient factor and dynamic pressure should be applied. The determination of the listed factors is not in the scope of this paper.

### 3.2. Sandwich Panel Subjected to Blast Load: Qualitative Response and Failure Mechanisms

**Figure 8:**
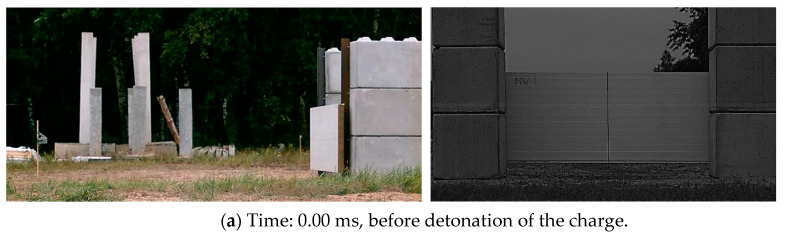

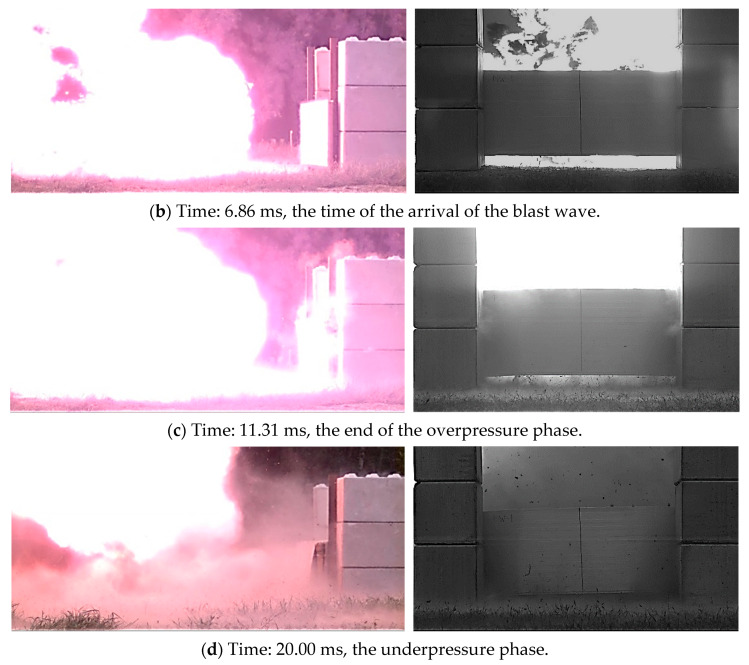
Selected frames recorded from Camera 1 (right side) and Camera (left side) for the following times: (**a**) 0.00 ms, before detonation of the charge; (**b**) 6.86 ms, the time of the arrival of the blast wave; (**c**) 11.31 ms, the end of the overpressure phase; and (**d**) 20.00 ms, the underpressure phase.

The kinematical response of the sandwich panels subjected to the blast load was recorded using high-speed cameras. The selected frames recorded from Camera 1 (right column) and Camera 2 (left column), for the specific times of the explosion, are presented in Figure 8. The vertical line at the backside of the panel was used to set the correct position for Camera 1.

**Figure 9:**
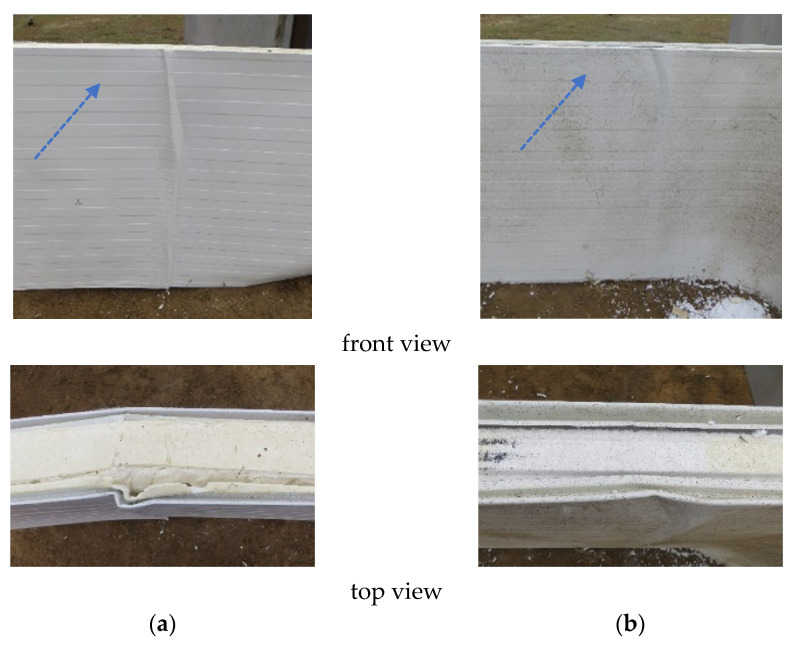
Wrinkling of the compressed facing (front and top view) for (**a**) the polyisocyanurate foam core and (**b**) the expanded polystyrene core.

The mechanical response of the sandwich panels subjected to the blast load (overpressure phase) revealed a number of failure mechanisms localized in the span of the panels and at the supports. In the span, the local buckling of the compressed facing (referred to in the literature as the wrinkling phenomenon [46,47]) was observed; see Figure 9.

**Figure 10:**
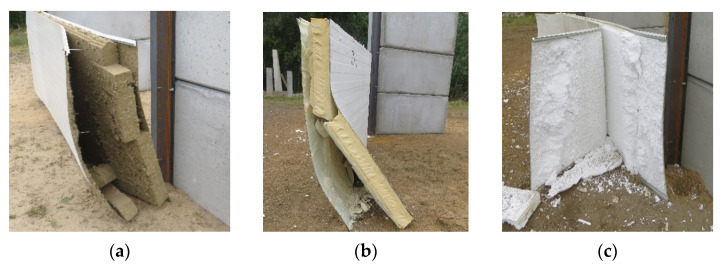
Comparison between the shear failure at the supports of the sandwich panels when using different core materials: (**a**) mineral wool; (**b**) polyisocyanurate foam; and (**c**) expanded polystyrene.

**Figure 11:**
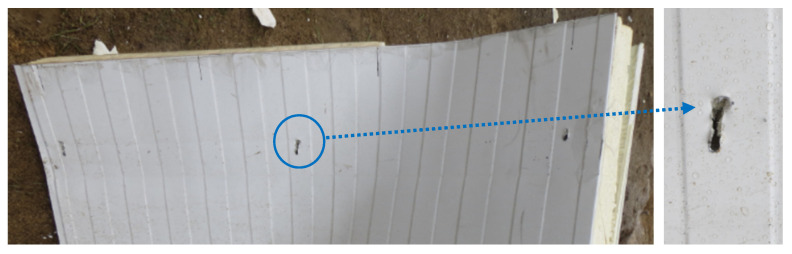
Tear failure of the internal facing in the vicinity of the fasteners.

**Figure 12:**
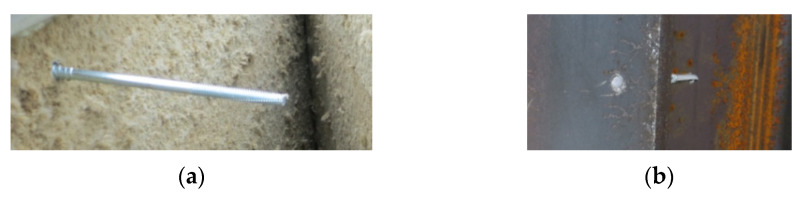
Shear failure of the fastener. (**a**) The upper part of the sheared fastener left in the sandwich panel’s external facing and (**b**) the lower part of the sheared fastener left in the steel column flange.

At the supports, shear failure of the core, tear failure of the internal facings in the vicinity of the fasteners, and shear failure of the fasteners were observed, shown in Figure 10, Figure 11 and Figure 12, respectively. Note that the core failure in the case of the noncontinuous material, represented in the research by the mineral wool, was propagated toward the middle of the panels. This caused the disturbance of the load transfer across the sandwich panel’s thickness. This was due to the lack of support of the facings by the core, which was damaged in the test. On the other hand, the core failure in the case of the continuous material (PIR foam, expanded polystyrene) was limited to the support area. This means that the integrity of the sandwich panel in the span of the experiment was not affected.

**Figure 13:**
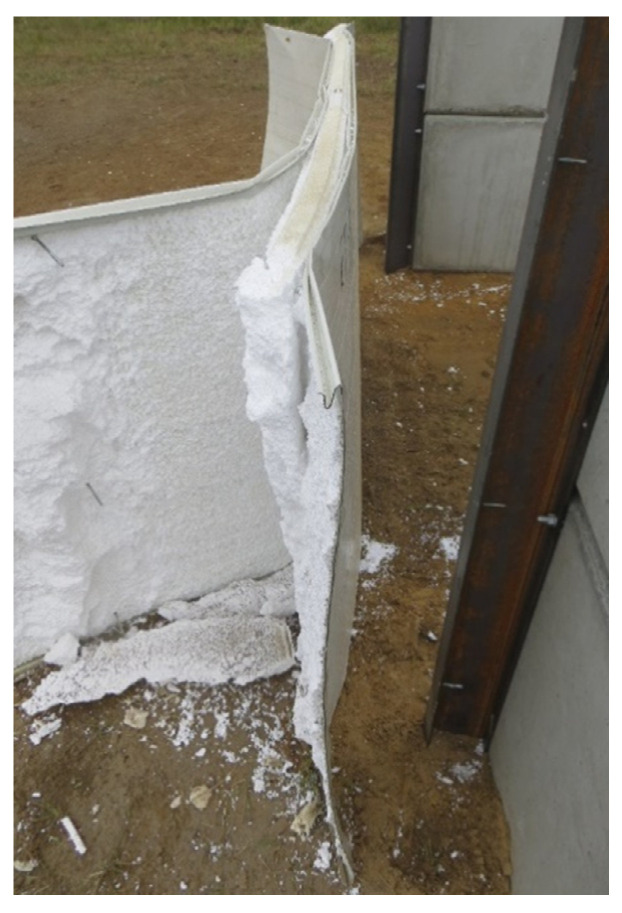
Displacement of the sandwich panel from the supporting structure.

Finally, during the underpressure phase of the blast load, the external facing was delaminated from the core layer and bent (Figure 10 and Figure 13), while the whole sandwich panel was moved from the supporting structure (Figure 13).

The evolution (sequence) of the failure mechanisms in the sandwich panels with continuous (polyisocyanurate foam, expanded polystyrene) and noncontinuous (mineral wool) core layers is presented in the next section.

## 4. Discussion

During the overpressure phase, the sandwich panels’ frontal facings (i.e., facing exposed to the blast wave) were subjected to normal compression stresses while the internal facing (rear facing) was under tension. The ultimate design for compression resistance of the facings includes local buckling, which in the literature is referred to as the wrinkling phenomenon. The design’s wrinkling strength (*f_swd_*) depends on the mechanical properties of the sandwich panel layer materials and can be expressed by the following relation: *f_swd_* = *η*_0_⋅(*G_C_*⋅*E_C_*⋅*E_F_*)^1/3^, where *η*_0_ is the reduction coefficient [46,47]. The value of the reduction coefficient depends on the geometrical and material assumptions made during the derivation of the formula for critical stresses. According to [47], the reduction coefficient *η*_0_ = 0.794; hence, the design’s wrinkling strength for a sandwich panel with a core made of polyisocyanurate foam, mineral wool, and expanded polystyrene equals 124.0 MPa, 88.0 MPa, and 75.0 MPa, respectively. The ultimate designed tension capacity of the facings is the design’s yield strength *f_yd_* of the material (steel grade S280; thus, *f_yd_* = 280.0 MPa). Yield failure of the tensioned facings was not observed for any of the tested panels, while wrinkling failure was obtained for the sandwich panels with cores made of polyisocyanurate foam and expanded polystyrene (continuous cores) (see Figure 9a,b, respectively). The limited size of the wrinkling failure in the case of the continuous core layers, as well as the lack of this failure in the case of the noncontinuous core layers, means that this type of failure was not the leading failure, but only the accompanying failure. The lack of wrinkling failure in the case of the mineral wool core layer was due to its lamellar structure. At the field test, it was observed that load transfer along the sandwich panel layers was disturbed in the panel with the mineral wool core due to the separation of the individual lamellas (see Figure 10a).

The leading failures of the tested sandwich panels were localized at the supports. The significant core failure (shear and crushing, Figure 10) resulted in both the tearing of the facings in the vicinity of the fasteners (Figure 11) and the shearing of the fasteners (Figure 12). Once the fasteners were sheared, the connection with the supports was lost.

## 5. Conclusions

This research dealt with the qualitative response and failure mechanisms of composite structures subjected to a blast loading scenario. The conducted tests revealed that the weakest links of a sandwich panel, subjected to a blast load, were both the fasteners and the strength of the core. Due to the shear failure of the fasteners, integrity between the sandwich panel (part of the cladding system of the buildings) and the main structure was not provided. This facade failure, from the point of view of the safety of the users, poses a threat to human life and health.

Future studies may test or look at the following aspects; the number of fasteners; the shear strength of the fasteners; the strength of the core at the supports, using the concept of core junctions [48,49]; the increased thickness of the facings at supports; and the shape of the longitudinal edge of the sandwich panel.

## Figures and Tables

**Table 1:** Selected industrial infrastructural elements, which are outside the building, and estimated basic data concerning their unconfined vapor cloud explosion (UVCE).

Infrastructural Element	Quantity (−)	Mass of Flammable Vapor Release *m_F_* (kg)	Maximum Pressure Developed at Completion of Combustion *P_max_* (kPa)	Blast Wave Energy *E* (kJ)	Equivalent of TNT Mass *W_TNT_* (kg)
propane cylinder 20l 30 bar	1	8	528.52	3807.8	0.82
	6	6 × 8 = 48		22,252.8	**4.95**
acetylene cylinder 20l 200 bar	2	2 × 4 = 8	989.69	3857.6	0.86
	12	12 × 4 = 48		23,145.6	**5.14**
hydrogen cylinder 0.75l 150 bar	1	9	858.75	15,839.9	3.52
	2	2 × 9 = 18		23,753.3	**5.28**
